# Global, regional and country burden of high BMI-related liver cancer among individuals aged above 70: trends from 1990 to 2021 and projections to 2044

**DOI:** 10.3389/fpubh.2025.1523578

**Published:** 2025-03-20

**Authors:** Ke-Jie He, Wanyi Shu, Yanggang Hong

**Affiliations:** ^1^The Quzhou Affiliated Hospital of Wenzhou Medical University, Quzhou People's Hospital, Quzhou, Zhejiang Province, China; ^2^School of Ophthalmology & Optometry, Wenzhou Medical University, Wenzhou, Zhejiang Province, China; ^3^The Second School of Medicine, Wenzhou Medical University, Wenzhou, Zhejiang Province, China

**Keywords:** liver cancer, age-standardized rate, BMI, GBD 2021, disability-adjusted life years

## Abstract

**Background:**

Liver cancer (LC) is a major global health concern, being the fourth leading cause of cancer-related mortality. Older adults are more susceptible, though mortality rates for those over 70 are declining. However, disability from non-communicable diseases remains high. High body mass index (BMI) is a notable risk factor for LC, with high BMI-related liver cancer (HB-LC) being a major concern.

**Methods:**

This study utilized Global Burden of Disease (GBD) 2021 dataset to assess the impact of HB-LC on individuals aged 70 and older from 1990 to 2021, with forecasts extending to 2044. Disease burden across socio-demographic index (SDI) regions was evaluated using age-standardized disability-adjusted life years (DALYs). Joinpoint regression and age-period-cohort models were used to analyze DALY trends and demographic influences, and decomposition analysis assessed the effects of population aging, growth, and epidemiological shifts.

**Results:**

Our findings revealed significant geographical disparities in HB-LC mortality, with East Asia, Southeast Asia, and parts of West Africa showing the highest rates. Global HB-LC DALYs increased by 2.49% annually, with low SDI regions experiencing recent acceleration. Gender disparity persisted, with males facing a steeper rise in HB-LC burden. Age-related DALY rates peaked in the 80–89 age groups, showing complex patterns across SDI regions. Epidemiological changes primarily drove the increased HB-LC burden in high SDI regions, while population growth was more significant in low SDI regions.

**Conclusion:**

This data analysis underscores necessity for region-specific public health strategies and demographic-focused interventions, enhancing surveillance and targeting efforts to mitigate the increasing burden of HB-LC among the older adults.

## 1 Introduction

Liver cancer (LC) has emerged as a global public health issue, ranking as the fourth leading cause of death and the sixth most prevalent cancer worldwide (1). Age is a major risk factor for liver cancer, with aging livers showing increased sensitivity to oncogenic stress (2, 3). The GBD 2019 study reveals that while overall mortality rates for those aged 70 and above have decreased, the burden of disability remains high, driven by non-communicable diseases (NCDs) and functional decline (4). LC contributes significantly to global mortality and disability-adjusted life years (DALYs). Understanding the etiologies and risk factors is essential for improving diagnostic accuracy and treatment outcomes (5, 6). Additionally, LC imposes a significant economic burden, encompassing both direct medical costs and indirect economic losses. Direct costs encompass expenses for diagnosis, treatment, and hospitalization, whereas indirect costs stem from labor loss and decreased productivity (7, 8).

The significant disease burden of LC is often linked to low socio-economic development, as well as limited access to and efficiency of healthcare systems (9, 10). Nonetheless, indicate that regions or countries with higher socio-economic status may experience a disproportionately high burden of LC due to lifestyle changes, metabolic disorders, and socio-economic factors (11, 12). Increasing prevalence of high body mass index (BMI) has become a notable risk factor for various cancers, including LC, in numerous nations (13, 14). High BMI-related liver cancer (HB-LC) is now widely recognized as a significant health issue, with excessive BMI contributing to LC-related fatalities (15, 16). Monitoring the trends of HB-LC, particularly among older populations, is critical for informing targeted public health measures.

The Global Burden of Disease (GBD) study is comprehensive epidemiological initiative that evaluates the worldwide effects of diseases, injuries, and risk factors (17). This study utilized the GBD 2021 dataset to evaluate the global and regional impact of HB-LC on older adult populations. It examined trends across different sociodemographic regions to identify patterns and disparities, providing insights for public health strategies to address this growing issue.

## 2 Materials and methods

### 2.1 Data source and disease definition

The primary source for investigating the burden of LC was the GBD study, available on the GHDx platform.^1^ Previous publications have carefully documented the scientific framework for the GBD 2021 study’s data merger, processing, and analysis (18–20). Utilizing the GBD 2021 dataset, our analysis examined LC deaths, DALYs, and age-standardized rates (ASRs) for individuals aged 70 and older from 1990 to 2021. This study focused on LC caused by HB-LC. Relevant International Classification of Diseases (ICD), 10th revision (ICD-10) codes (C22–C22.4 and C22.7-C22.8), was employed to identify HB-LC cases. GBA 2021 provided 95% uncertainty intervals (UIs) (21).

### 2.2 Sociodemographic index

The 2015 IHME evaluation emphasized connection between population health and social development globally. It calculated the total fertility rate for those under 25 years of age based on education levels of individuals aged 15 years and older, with per capita income adjusted for lag and scored from 0 to 1. In GBD 2021, SDI values ranged from 0 (lowest income, education, fertility) to 100 (highest). The 204 nations and territories were divided into five SDI levels: low, low-middle, middle, high-middle, and high, enabling detailed analysis of regional disparities in LC’s economic development impact (20).

### 2.3 Age-standardized DALYs

DALYs combine years of life lost (YLLs) and years lived with disability (YLDs) to measure disease burden, as per GBD methodology. They quantify health loss from illnesses and injuries, considering deaths, mortality (22). For a better comparison between nations and SDI regions, age-standardized DALYs per 100, 000 inhabitants were used to calculate global problem of HB-LC in this study to compare 1990 to 2021.

### 2.4 Statistical analysis

We used statistical methods to analyze HB-LC burden, including joinpoint regression for trends in DALYs from 1990 to 2021, age-period-cohort analysis for age and period trends, and decomposition analysis to assess the impact of aging, growth, and epidemiological changes across SDI regions. The Nordpred model forecasts the global HB-LC burden for those over 70, by 5-year age groups and gender, until 2044. Model Parameters: The Nordpred model was applied using 5-year age groups (70–74, 75–79, 80–84, 85–89, 90–94, and 95+ years) and gender-specific data (male and female) for the period 1990–2021. The model incorporates age, period, and cohort effects to project future trends in high BMI-related liver cancer (HB-LC) burden. Data Inputs: The model utilized age-standardized disability-adjusted life years (DALYs) and mortality rates from the Global Burden of Disease (GBD) 2021 dataset as primary inputs. Projection Assumptions: The projections assumed that current trends in HB-LC incidence, mortality, and DALYs would continue, accounting for population aging, growth, and epidemiological changes. Validation: The model’s accuracy was validated by comparing projected values with observed data for the most recent years (2015–2021), ensuring reliability in the forecasts. GBD 2021 adheres to GATHER for accurate and transparent health estimates (23). Analyses utilized R (v.4.1.0), joinpoint regression software (v.4.6), and STATA (v.15.1). A *p*-value threshold of 0.05 was used to determine statistical significance. Detailed methodologies are provided in the Supplementary methods.

## 3 Results

### 3.1 HB-LC deaths and DALY global burden in 2021

**Figure 1:**
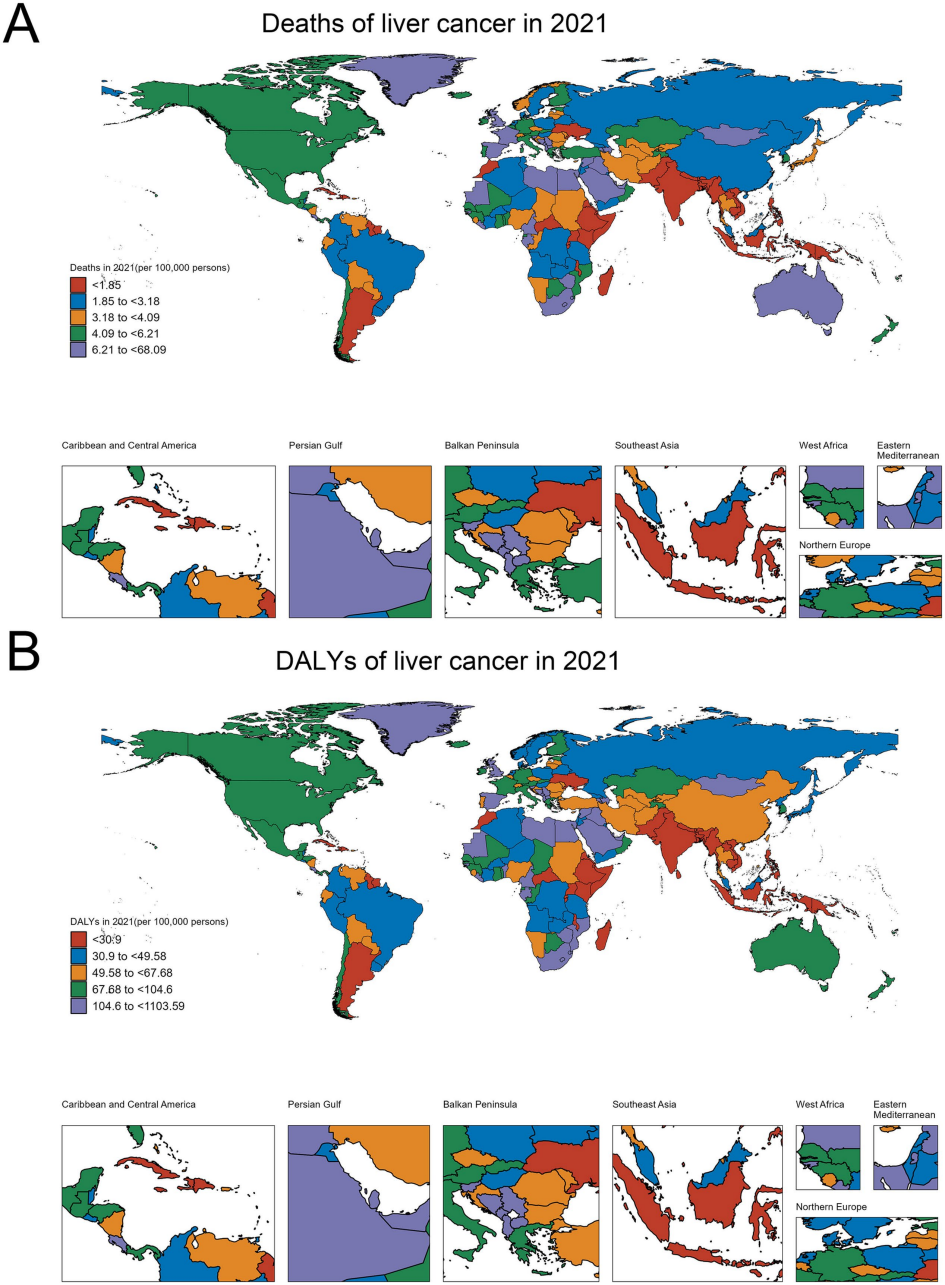
Global distribution of HB-LC burden in 2021. **(A)** Deaths per 100,000 persons. **(B)** Age-standardized DALYs per 100,000 persons.

Analysis of High BMI-related liver cancer (HB-LC) burden in 2021 revealed significant global variations and distinct regional patterns across 204 countries and territories (Figure 1; Table 1; Supplementary Table S1; Supplementary material 1). The East Asia and Pacific region, particularly Mongolia, showed the highest rates globally (death rate: 68.087, DALY rate: 1103.586), followed by countries in the Middle East and North Africa, such as Egypt (death rate: 33.998, DALY rate: 583.520), Qatar (death rate: 33.135, DALY rate: 562.666), and the United Arab Emirates (death rate: 22.244, DALY rate: 415.002). While Sub-Saharan Africa generally exhibited lower rates, some countries like Eswatini (death rate: 24.155, DALY rate: 410.744) and Zimbabwe (death rate: 13.579, DALY rate: 240.922) showed unexpectedly high burdens. High-income countries like the United States (death rate: 5.971, DALY rate: 95.851) and Japan (death rate: 3.476, DALY rate: 47.343) demonstrated moderate burdens relative to the global range. The mean DALY to death ratio of 16.33ncluded Timor-Leste (death rate: 0.145, DALY rate: 2.452), Mauritius (death rate: 0.417, DALY rate: 6.805), and Ethiopia (death rate: 0.460, DALY rate: 7.919).

**Table 1:** Death and DALY rates of HB-LC among individuals aged 70+ in selected countries in 2021 (Data source: Supplementary Table S1).

Country	Death rate (per 100,000)	DALY rate (per 100,000)
Tonga	22.004 (8.077–41.385)	359.916 (131.372–678.715)
Fiji	5.246 (2.155–9.172)	88.714 (36.547–156.376)
Mongolia	68.087 (27.459–120.451)	1103.587 (441.747–1964.627)
United States of America	5.971 (2.456–9.926)	95.851 (39.847–158.963)
Egypt	33.998 (14.017–57.184)	583.520 (239.635–999.153)
Japan	3.476 (1.450–5.760)	47.343 (19.645–76.853)
Nigeria	3.887 (1.478–6.480)	62.711 (23.692–104.090)

Death rates and DALY rates showed a strong positive correlation (*r* = 0.98, *p* < 0.001), indicating aligned mortality and overall disease burden patterns. The mean DALY to death ratio of 16.33 (SD = 0.93) suggests that each HB-LC death corresponded to about 16 years of healthy life lost. Notably, some countries, particularly small island nations such as Tonga (death rate: 22.004, DALY rate: 359.916) and Fiji (death rate: 5.246, DALY rate: 88.714), demonstrated disproportionately higher ratios relative to their size and regional context, highlighting significant health challenges in these regions.

### 3.2 Temporal trends in age-standardized HB-LC DALYs by SDI regions (1990–2021)

**Figure 2:**
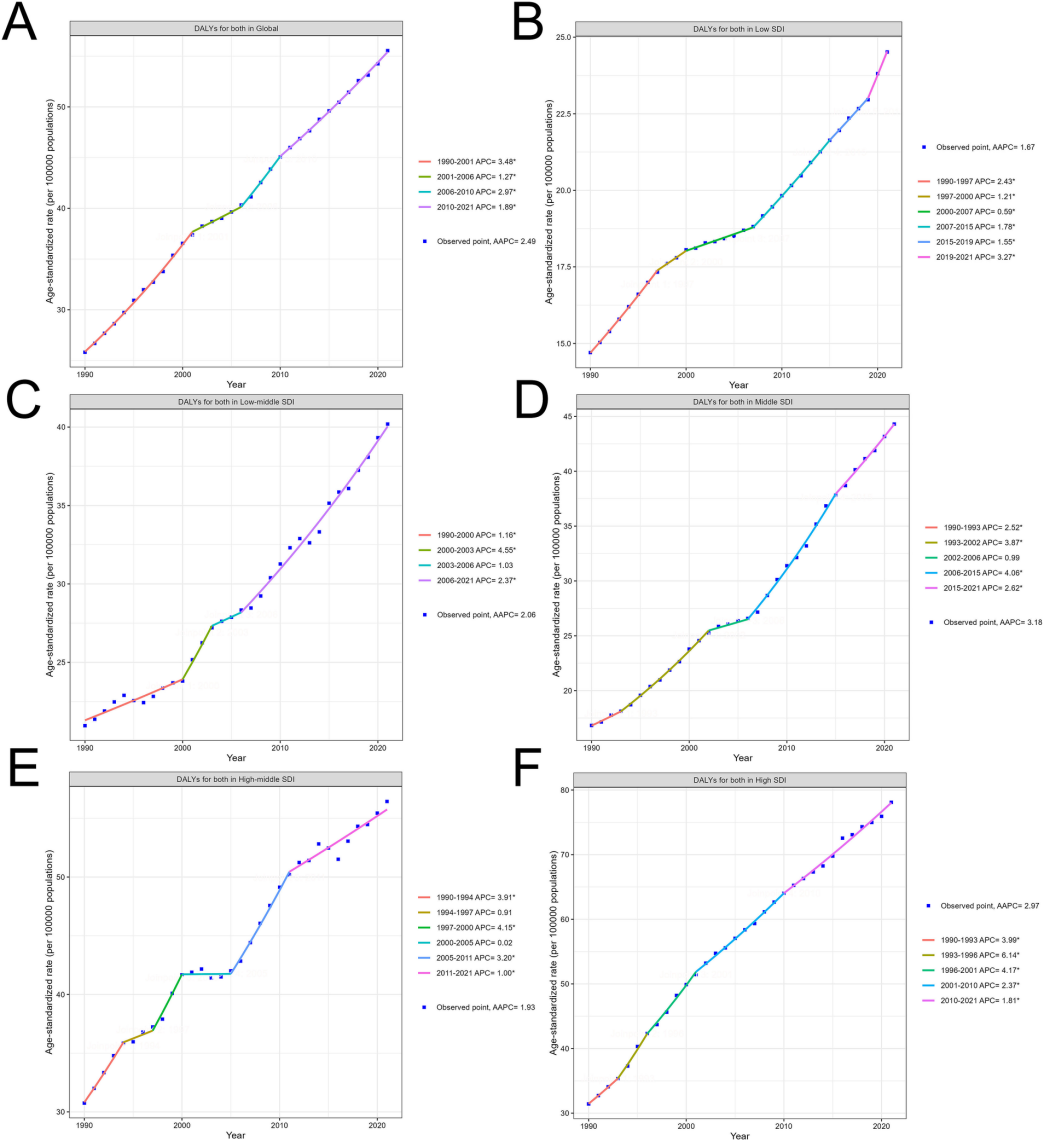
Trends in age-standardized HB-LC DALYs by SDI Regions (1990–2021). **(A)** Global, **(B)** Low SDI, **(C)** Low-Middle SDI, **(D)** Middle SDI, **(E)** High-Middle SDI, and **(F)** High SDI regions. AAPC and significant APC periods are indicated for each region.

Across all SDI regions, HB-LC-related DALY rates consistently increased from 1990 to 2021, with variations in magnitude and patterns of growth (Figures 2A–F; Supplementary material 2; Supplementary Table S2). Average annual percentage change (AAPC) for the entire period was highest in Middle SDI regions at 3.18% (95% CI: 2.97 to 3.38), followed by high SDI regions at 2.97% (95% CI: 2.75 to 3.20), and low SDI regions at 1.67% (95% CI: 1.60 to 1.74). High SDI regions maintained the highest absolute DALY rates, increasing from 2.5 per 100,000 in 1990 to 10.3 per 100,000 in 2021, while middle SDI regions showed the most rapid growth, rising from 1.8 to 9.3 per 100,000. Low SDI regions increased from 1.0 to 5.9 per 100,000 over the same period.

Joinpoint analysis revealed distinct periods of change for each SDI region. High SDI regions experienced four joinpoints, with the highest Annual percentage change (APC) of 6.14% (95% CI: 4.16 to 8.14) between 1993 and 1996. Middle SDI regions showed four joinpoints, with the highest APC of 4.06% (95% CI: 3.82 to 4.31) occurring between 2006 and 2015. Low SDI regions demonstrated five joinpoints, with relatively stable APCs throughout the period, ranging from 0.59 to 3.27%. In the most recent period (2015–2021), APCs were 1.81% (95% CI: 1.69 to 1.94) for high SDI, 2.62% (95% CI: 2.23 to 3.00) for middle SDI, and 3.27% (95% CI: 2.76 to 3.79) for low SDI regions, suggesting a potential slowing of growth in high SDI regions, while middle and low SDI regions continue to show concerning increases.

### 3.3 Time trends in HB-LC DALY rates among older adult individuals

**Figure 3:**
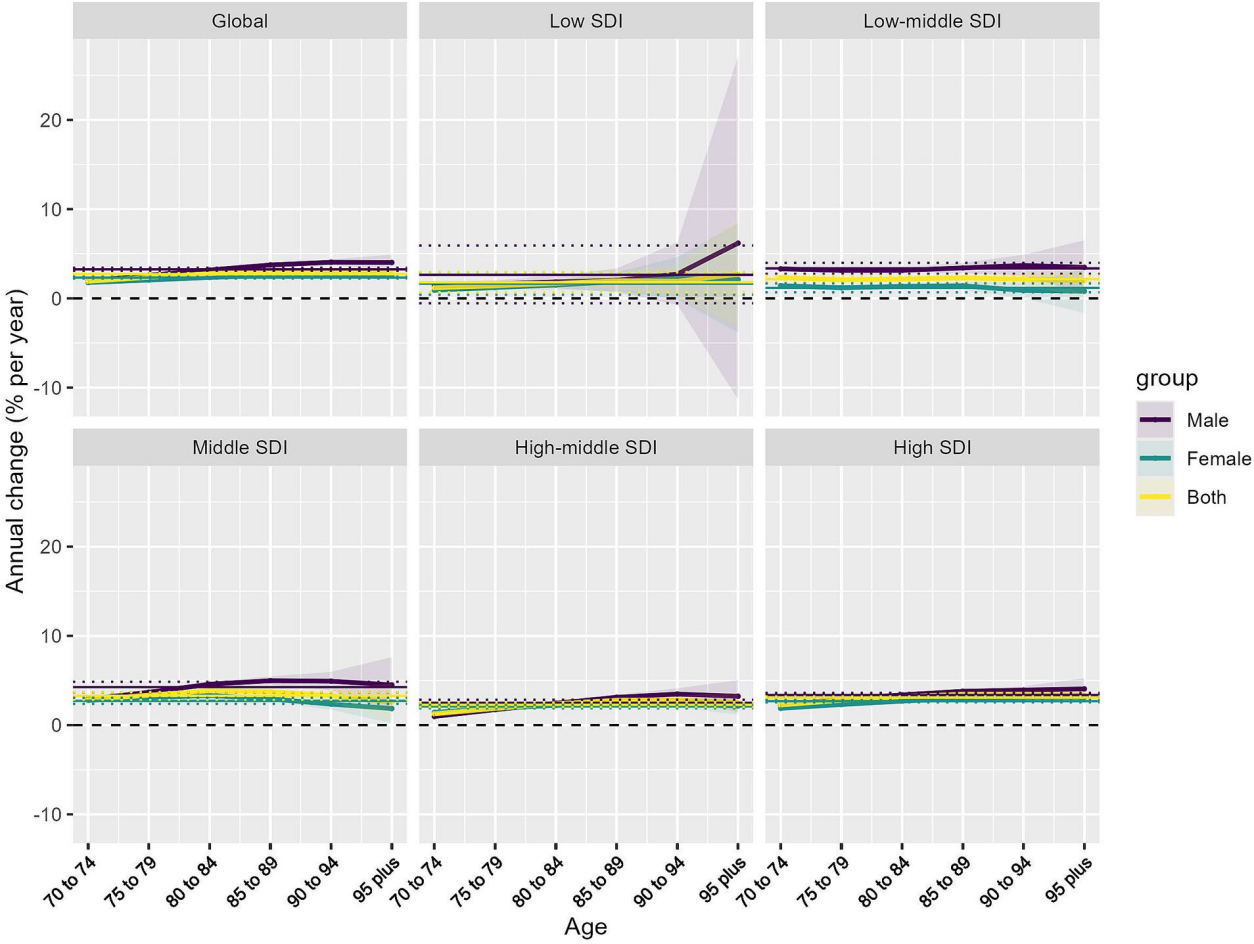
Local drifts of HB-LC DALYs for individuals aged 70 and above, 1990–2021, stratified by SDI region and sex. Lines represent trends for males, females, and both sexes combined.

Analysis of yearly HB-LC DALY rate changes in individuals aged 70 and above revealed consistent increases across all SDI regions, as evidenced by net drift analysis (Supplementary material 3; Supplementary Table S3) and local drift (Figure 3) patterns. Globally, the net drift for males (3.25% per year, 95% CI: 3.08–3.42%) significantly outpaced that of females (2.33% per year, 95% CI: 2.23–2.43%), indicating a more rapid increase in HB-LC burden among older adult men. Regional disparities were notable, with middle SDI regions showing the highest net drift for males (4.26% per year) and high SDI regions leading for females (2.68% per year). Low SDI regions showed the slowest HB-LC burden progression for both genders. Local drift analysis indicated age-specific variations across SDI regions, with positive drift values increasing with age, especially in middle to high SDI regions, peaking at 85+. In contrast, low-middle and low SDI regions had more uniform age patterns, with smaller differences between the 70–74 and years and 85+ years cohorts.

Gender disparities were evident across all SDI regions and age groups, with males, particularly in middle and low-middle SDI regions, exhibiting higher local drift values than females. The higher increase rates in more developed regions, especially among males and older age groups, indicate that aging-related factors in these societies may significantly contribute to the rising HB-LC burden.

### 3.4 Impact of age and period on HB-LC DALYs

**Figure 4:**
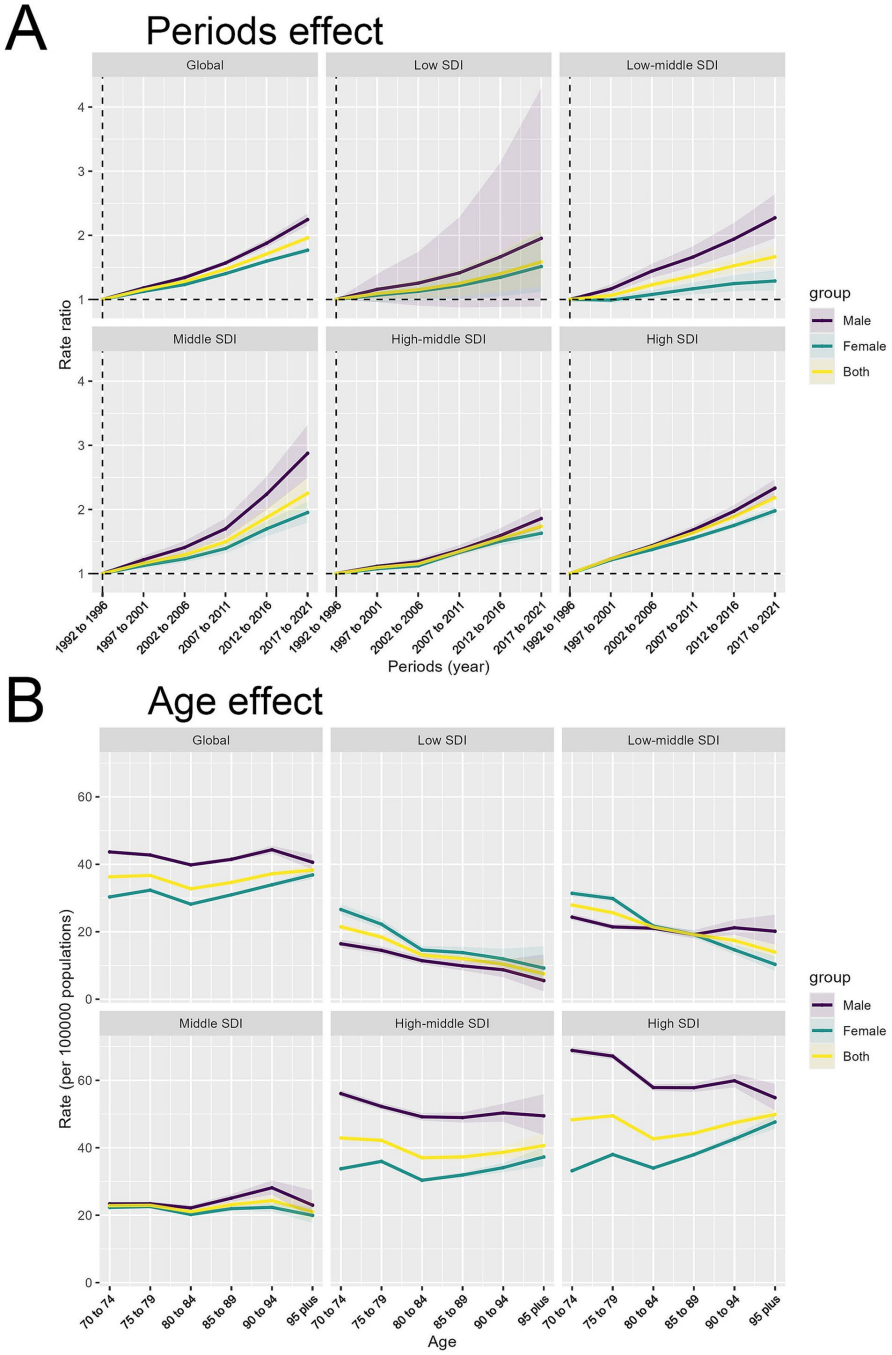
Period and age effects on HB-LC age-standardized DALYs by SDI region and sex. **(A)** Period effects showing rate ratios over time. **(B)** Age effects depicting rates per 100,000 population by age group.

Period effects (Figure 4A) illustrated a global rise in rate ratios (RRs) from 1990 to 2021 across all regions, with distinct patterns based on SDI levels. Globally, RRs increased steadily for both genders, with a sharp rise in low SDI regions, notably among males, highlighting health disparities. Low-middle and middle SDI regions also saw significant growth, with middle SDI regions showing a marked recent increase. High-middle and high SDI regions experienced more gradual increases and smaller gender disparities.

The analysis of DALY rates per 100,000 population demonstrated intricate age-related patterns across different SDI regions (Figure 4B). Globally, DALY rates are stable across ages, with males generally higher, peaking at ages 80–89. Low SDI regions have lower rates and minimal gender differences. Low-middle SDI regions have slightly higher rates, especially at 70–79 years. Middle SDI regions show less decline and maintain gender disparities. High-middle SDI regions have higher rates with a clear male predominance, while high SDI regions demonstrate the most significant gender disparities, characterized by substantially higher rates among males, particularly among older individuals. Although DALY rates typically rise with age, they decrease in older age groups within low and low-middle SDI regions, unlike the patterns seen in higher SDI regions.

### 3.5 Decomposition of factors contributing to HB-LC burden (1990–2021)

**Figure 5:**
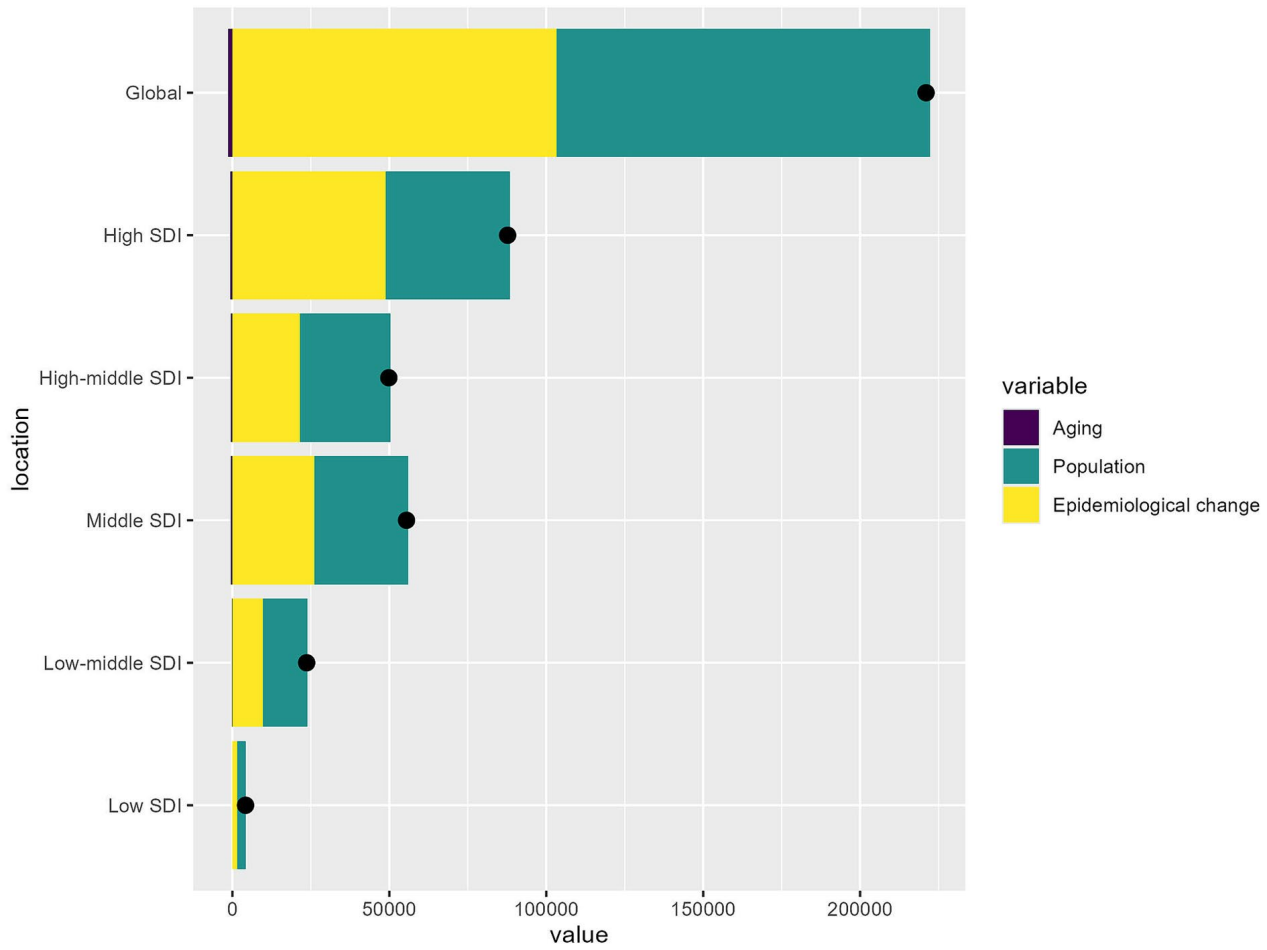
The relative contributions of aging, population growth, and epidemiological changes to the increase in HB-LC DALYs from 1990 to 2021 across different SDI regions.

Figure 5 analyzed the factors affecting HB-LC burden shifts across SDI regions from 1990 to 2021, focusing on epidemiological changes, population aging, and growth. Globally, population growth was the main contributor to the HB-LC burden, followed by epidemiological changes, while population aging had the least impact. In high SDI areas, population growth was the main factor, with notable contributions from epidemiological changes, while aging had a lesser but more significant impact than in lower SDI regions. In high-middle and middle SDI regions, population growth slightly exceeded epidemiological changes. In low-middle SDI regions, both factors had equal influence, with aging having little effect. In low SDI regions, all factors minimally impacted the burden change, though population growth was slightly more influential.

### 3.6 Projected global trends in HB-LC burden for individuals aged 70 and above (2021–2044)

**Figure 6:**
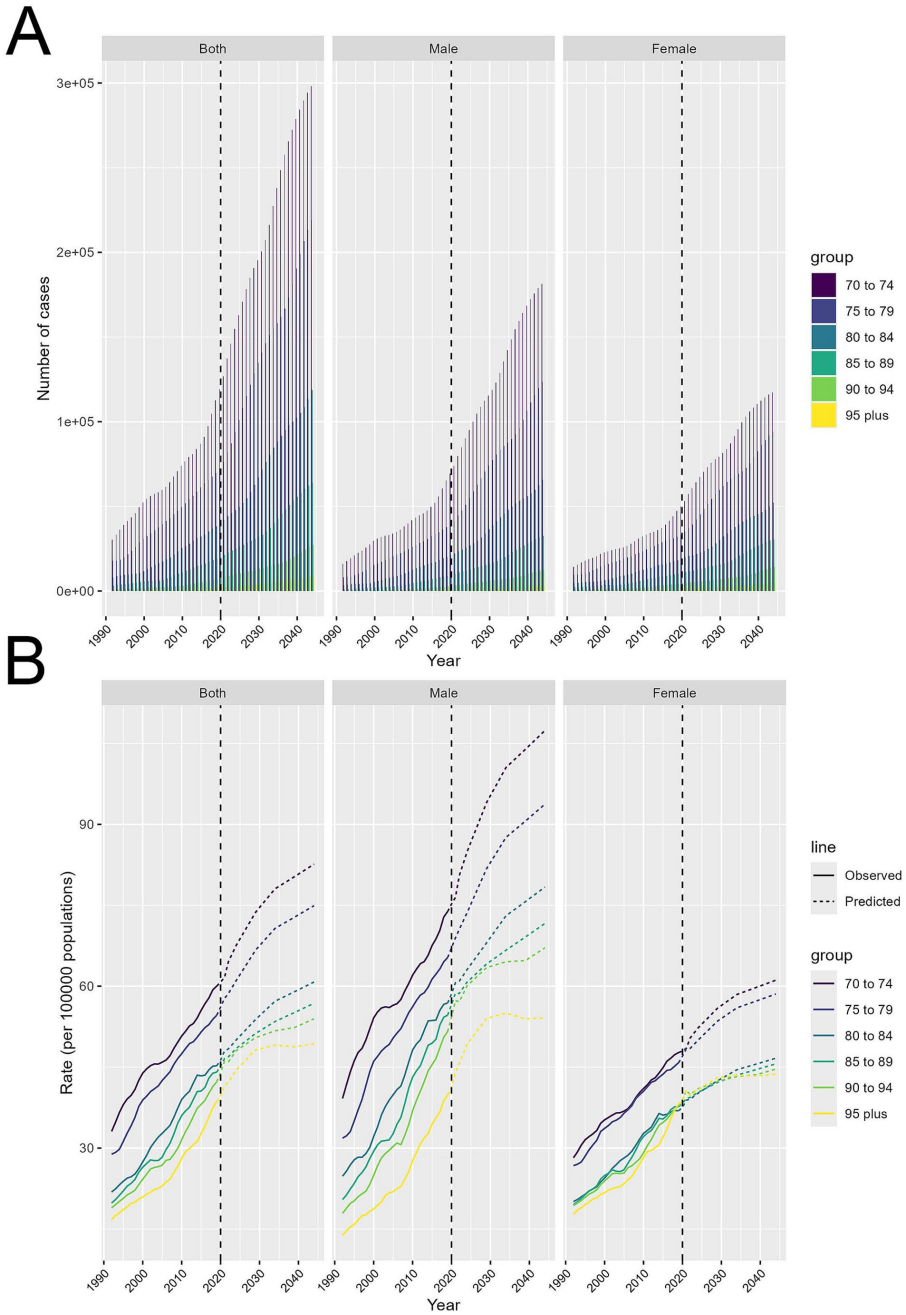
Projected trends in HB-LC burden for individuals aged 70 and above from 2021 to 2044, stratified by age cohorts and gender. **(A)** Number of cases. **(B)** Age-standardized DALY rates per 100,000.

HB-LC cases are set to rise significantly for both genders (Figure 6A). Cases have been rising since 1990 and are projected to continue increasing until 2044, especially among those aged 70–79. Both sexes show a steady rise, but males experience a sharper increase, particularly in the 70–79 age range. Females also see growth, though less steep, with the 70–74 and 75–79 age groups having the highest numbers.

Age-standardized DALY rates per 100,000 show differing trends (Figure 6B). DALY rates are consistently rising across both sexes, especially in the 70–74 and 75–79 age groups, which are expected to have the highest rates through 2044. Older groups, like 90–94 and 95+, show a slower increase. Males experience a faster rise in DALY rates than females, particularly in the 70–74 and 75–79 age groups. Females follow a similar pattern, but their rate increase is less pronounced.

## 4 Discussion

LC remains a significant global health issue, and high BMI is increasingly recognized as a major risk factor driving its incidence and mortality (24). This study utilized GBD 2021 data to investigate HB-LC in individuals aged 70 and above, examining disease burden by age, sex, and SDI through DALYs, with an emphasis on BMI and the older adults to provide new insights into LC impacts.

In 2021, global HB-LC death rates varied widely from 0.145 to 68.87 per 100,000 people, highlighting the uneven impact of high BMI on HB-LC. Mongolia’s rates are especially concerning due to widespread hepatitis B and C and increasing obesity (25). East Asia and the Middle East constantly exhibited significant contributions. Also, high levels in Middle Eastern countries like Egypt, Qatar, and the United Arab Emirates may be affected by rapid urbanization and Western dietary habits, connecting with tendencies reported in 2023 (25). The strong link between high BMI and increased mortality and DALY levels emphasizes its global impact on health. In high-income countries like the U.S. and Japan, moderate HB-LC burdens suggest that factors beyond wealth, such as healthcare efficiency, public health policies, or genetics, play a role. Meanwhile, some low-income Sub-Saharan nations report lower costs, likely due to underreporting or limited testing rather than an actual lower disease burden (26). In these regions, resource constraints frequently cause underdiagnosis of NCDs (27). While DALY to death ratios vary, some countries experience more significant quality of life impacts. HB-LC growth is shaped by genetics and environment, including diet and lifestyle. Small island nations like Tonga and Fiji face unique challenges, as lifestyle changes, particularly higher consumption of high-fat and processed foods, significantly increase NCDs (28).

From 1990 to 2021, HB-LC DALYs increased across all SDI regions, aligning with previous research suggesting that the rising prevalence of obesity is a critical driver of global cancer burden (29–31). High SDI regions had slower growth rates, likely due to obesity prevention and better liver cancer screening, while middle and low SDI regions saw significant increases, highlighting the urgent need for proactive public health measures, possibly due to a rapid shift to energy-dense diets and sedentary lifestyles outpacing public health efforts (32, 33). Meanwhile, the lower but steady increase observed in low SDI regions suggests an emerging problem that could escalate in the future (34).

The observed gender disparity in HB-LC burden, with males experiencing a steeper rise globally, warrants further investigation. This trend may be influenced by several factors: higher rates of smoking and alcohol use, greater impact of high BMI on LC risk, the carcinogenic effects of androgens, and delayed healthcare-seeking behaviors in men (8, 35–37), more highlighting the importance of investigating gender- certain risk factors and interventions. The higher incidence of HB-LC in older males is due to several factors. Testosterone increases liver fat and oxidative stress, leading to liver disease and cancer (38). Men also store more visceral fat, linked to insulin resistance and inflammation (39). Behavioral factors like higher alcohol and tobacco use, combined with obesity, further elevate their risk (40). Women, on the other hand, benefit from estrogen’s liver-protective effects, which may reduce their risk (41). Men are also less likely to seek preventive healthcare, delaying liver disease diagnosis (38). Additionally, genetic and epigenetic differences between sexes may affect vulnerability to liver diseases related to obesity (42).

Age-related DALY rates for HB-LC show complex patterns across SDI regions, influenced by socioeconomic factors, healthcare systems, and demographic changes. Globally, DALY rates peak in the 80–89 age groups, indicating a significant burden among older adults (43). Targeted strategies must be employed to combat the effects of HB-LC due to the aging population and altering eating habits (30). Differences in age-related DALY patterns across SDI regions reveal healthcare disparities and life expectancy variations. In lower SDI regions, DALY rates drop significantly after ages 75–79, likely due to limited healthcare access and shorter life expectancy (15). In contrast, higher SDI regions show more stable or even increasing rates in older age groups, likely reflecting better healthcare systems and longer life expectancies (44). Males face a higher and faster-growing HB-LC burden, highlighting the need for targeted interventions, especially for men and the very older adults. The paradox of higher DALY rates among males aged 80–89 in high-SDI regions, despite declining androgen levels with age, warrants closer investigation. This can be explained by factors such as lifelong exposure to liver cancer risks like alcohol, hepatitis, and metabolic disorders (6, 45). Better healthcare in these regions may lead to longer survival of liver cancer patients, increasing DALY rates (46). A cohort effect may also exist, as this age group might have faced higher hepatitis B exposure before vaccination programs (47). Additionally, the rise of NAFLD and NASH in older populations, especially in high-SDI areas with high obesity rates, could worsen this trend (48). Lastly, a survivor effect might be at play, with men in high-SDI regions who reach advanced age possibly having genetic or lifestyle traits that both enhance longevity and elevate liver cancer risk (49).

Several factors contribute to the increasing HB-LC burden among individuals aged 70 and above. Epidemiological shifts, including the increasing prevalence of non-alcoholic steatohepatitis and obesity-related metabolic syndromes. Demographically, global population aging and sociodemographic shifts in regions with higher sociodemographic indices exacerbate the burden (50). Regional variations, influenced by factors such as urbanization and lifestyle changes, further complicate the trend (48). Urban environments like Shanghai are experiencing changes in cancer patterns, particularly an increase in liver metastases from colorectal and gastric cancers (51). Epidemiological factors are more crucial in high SDI areas, whereas demographic factors like population growth matter more in low SDI regions, indicating a need for region-specific interventions.

Our projections from 2021 to 2044 indicate a sustained increase in HB-LC cases, especially among those aged 70–79. This aligns with global trends showing a rise in LC and its disparities (52). Our research offers new insights into HB-LC, emphasizing the significant role of high BMI in LC, which is increasingly recognized as a major cancer risk factor (53). We noted a significant increase in HB-LC cases among males, aligning with LC trends. The expected rise in age-standardized DALY rates, especially in the 70–79 age group, highlights the escalating impact of obesity and metabolic disorders on LC. Recent studies also link metabolic syndrome and obesity to higher cancer risk (53). Our research indicates that aging populations and increasing obesity rates significantly impact HB-LC burden, surpassing traditional factors like viral hepatitis (52). Addressing metabolic risk factors could change the projected HB-LC burden, providing a new approach to LC prevention amid global obesity trends.

Despite a thorough analysis, our study has limitations that should be acknowledged. While we focused on high BMI as a significant risk factor for HB-LC, liver cancer is a multifactorial disease influenced by various other factors. Key confounders such as HBV/HCV co-infections, diabetes, and alcohol use were not explicitly controlled for in this analysis. Data from the GBD database may be unreliable in low-SDI regions due to weaker healthcare systems, potentially causing underreporting or misclassification of HB-LC cases and deaths. The study used age-standardized DALY rates and incidence to assess HB-LC burden but ignored key risk factors like alcohol use, viral hepatitis, and genetics. High BMI is significant, but LC involves multiple factors, and ignoring these limits full comprehension. The observed decline in DALY rates for people aged 80+ in low-SDI regions may be due to selective survival bias, where healthier individuals live longer while those with severe health issues die younger due to limited healthcare. This survivor effect could skew the DALY rates for older age groups. Additionally, underreporting and misclassification of HB-LC cases in these regions, due to weaker healthcare systems, may also influence this trend. Additionally, the GBD’s CODEm algorithm cannot fully resolve the misattribution of metastatic liver cancers to HB-LC, which may lead to overestimation or misclassification in certain cases. Furthermore, the 20-year latency period between obesity onset and HCC development is unaccounted for in our analysis, potentially impacting the accuracy of burden estimates. Emerging therapies (e.g., GLP-1 agonists), which may reduce obesity prevalence, are not accounted for, and their long-term hepatic impacts remain uncertain. Thus, these projections should be approached cautiously. Lastly, while the study offers a global perspective, it may not capture regional differences in healthcare, culture, and socioeconomic factors affecting LC outcomes. Future research should include local data to better understand regional influences and support targeted interventions.

## 5 Conclusion

This study on HB-LC in individuals over 70 reveals global disparities and trends from 1990 to 2021, with projections to 2044. It highlights the complex impact of epidemiological, demographic, and healthcare factors across SDI regions, showing variations in DALY rates by geography, SDI, gender, and age. The findings emphasize the need for region-specific public health strategies, including better surveillance, targeted obesity prevention, improved healthcare access, and international collaboration.

## Data Availability

The original contributions presented in the study are included in the article/Supplementary material, further inquiries can be directed to the corresponding author.

## Footnotes

^1^ https://vizhub.healthdata.org/gbd-results/
