# Supplementary material for: Global, regional and country burden of high BMI-related liver cancer among individuals aged above 70: trends from 1990 to 2021 and projections to 2044
Source: Front Public Health. 2025 Mar 20;13:1523578. doi: 10.3389/fpubh.2025.1523578 (PMC11965621; doi:10.3389/fpubh.2025.1523578)
Supplement: Supplementary file 1 [file Supplementary_file_1.docx]

**Supplementary Methods**

**S1. Joinpoint analysis**

We used Joinpoint regression models to examine the developments in disability-adjusted life years (DALYs) related to high BMI-related liver cancer (HB-LC) between 1990 and 2021 to determine important historical shifts in the problem of liver cancer (LC). The horizontal slope of DALY price trends over time was significantly altered by this scientific approach [1]. We calculated the Annual Percentage Change (APC) to account for year-to-year variations for each identified trend segment. The Average Annual Percentage Change (AAPC) with 95% confidence intervals (CIs) was used to analyze overall trends during the study period [2, 3]. To enable a thorough analysis of trends across different demographic and economic subgroups, analyses were performed by SDI areas, age groups, and gender.

The Joinpoint regression model, created by the National Cancer Institute's Division of Cancer Control and Population Sciences, is extensively used in analyzing trends in disease incidence and mortality rate [4]. For our analysis of HB-LC DALYs, we utilized the logarithmic linear model.

Joinpoint regression analysis includes two main models: the linear model (y = xb) and the log-linear model (ln y = xb). When the dependent variable approximates a normal distribution and the sample size is substantial (typically exceeding 100), the linear model is generally preferred. This is often the case for continuous variables such as height or weight. Conversely, the log-linear model is more appropriate when the dependent variable follows an exponential or Poisson distribution. Specifically, We set the minimum number of joinpoints to 0 and the maximum to 3, with a significance level of 0.05. The grid search method was employed for identifying optimal joinpoints.

Joinpoint's default modeling approach utilizes the grid search method (GSM). This technique partitions the study data into a grid, with each intersection representing a potential scenario. It then calculates performance metrics for corresponding equations at each point within specified intervals, using a predetermined step size to identify the optimal function. Essentially, the Joinpoint model employs GSM to establish all possible segment function breakpoints (i.e., Joinpoints) and computes the sum of squares errors (SSE) and mean squared errors (MSE) for each potential scenario. The model selects the grid point with the lowest MSE as the Joinpoint for the segment function and subsequently fits equation parameters such as β_0_, β_1_, δ_1_, ..., δ_k_ based on the chosen Joinpoints and interval functions [5].

The Joinpoint model primarily utilizes two key outcome indicators: APC and AAPC, along with their respective 95% CI. As its name implies, APC reflects the yearly percentage variation in the dependent variable. For instance, in a log-linear model expressed as ln(y) = β_0_ + β1x, where y denotes the incidence rate and x represents the year of occurrence, the formula for computing APC in the fitted model can be derived as follows:


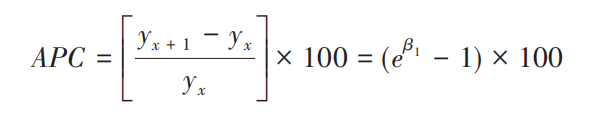


The 100 (1-α)% confidence interval is defined by its lower and upper limits.


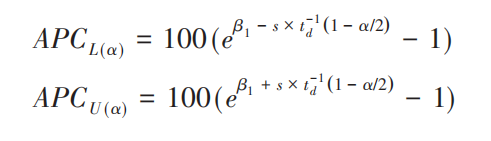


In the aforementioned equation, β_1_ denotes the regression coefficient, s represents β_1_'s standard error, d signifies the degrees of freedom, and t_d_(q) corresponds to the qth percentile of the t-distribution with d degrees of freedom (e.g., 95%).

APC is employed to assess the internal trend within each independent interval of a segmented function or the overall trend without connecting points. However, when evaluating the average change trend across multiple intervals, AAPC becomes necessary. The AAPC parameter calculation method involves a weighted computation of regression coefficients for each interval, based on the segment interval width w. Its formula is expressed as:


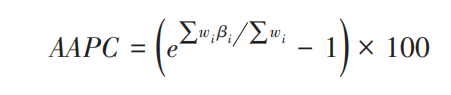


The lower and upper limits of the 100(1-α) % confidence interval are respectively:


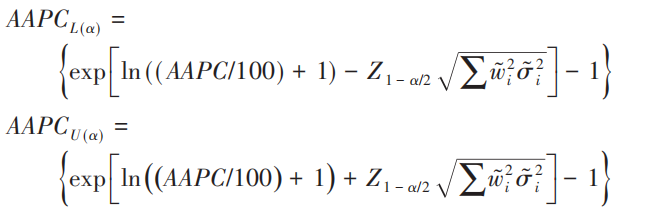


In the equation above, w_i_ signifies the breadth of each segment function interval (i.e., the number of years encompassed), β_i_ represents the regression coefficient for each interval, σ^2^i denotes the variance of β_i_, and Z_α_ corresponds to the α percentile value in the normal distribution.

To acquire the Joinpoint software, we accessed the National Cancer Institute's website, created an account, and submitted our application details.

**S2. Age-period-cohort analysis of HB-LC DALYs**

HB-LC stress trends were analyzed based on age, period, and birth demographic [6]. Age-period-cohort model incorporates biological aging, technological, and social factors alongside traditional epidemiological analyses [7]. Age-period-cohort models are implemented using freely available R tools, with methodological details provided in prior publications [8]. In this review, we divided the HB-LC DALY data into six-time intervals and six-year intervals respectively. To capture demographic and socioeconomic variations, data were categorized according to SDI regions (low, low-middle, middle, high-middle, and high), as well as gender (male, female, and combined). Net drift represents the overall annual percentage change in DALY levels for each time group. Local drifts reflect annual percentage changes by period for each age group. To compare age-specific rates for each period to the reference period (1992-1996), we created rate ratios (RRs). To determine the age effect, we utilized longitudinal age curves to represent DALYs rate per 100, 000 people across a range of age groups.

The APC model utilized GBD 2021 DALY estimates for HB-LC alongside population data from each country/region. In a typical APC model, age and period intervals are uniformly divided, often into five-year segments. To standardize GBD estimates, we reorganized the data into a uniform framework by choosing DALY and population figures from the midpoint of each five-year interval (e.g., [1994] 1992–1996, [1999] 1997–2001, ... [2019] 2017–2021) to represent each period. The input data encompassed 6 age groups (from 70-74 to 95+ in five-year intervals). The input data encompassed 6 age groups (from 70-74 to 95+ in five-year intervals). The APC model calculated the annual percentage change (net drift, % per year) to represent the overall temporal trend in HB-LC DALYs. The net drift is influenced by two components: the trend related to calendar time and the trend associated with age-specific changes. The model assessed the temporal trend of DALYs for each age group, represented as the annual percentage change in age-specific DALYs (local drift, % per year), indicating age-related trends. A drift of ±1% annually is deemed significant for DALYs, as it corresponds to changes of approximately ±10%, ±18%, and ±26% in the fitted rate over 10, 20, and 30 years, respectively. A Wald chi-squared test [9] was used to assess the significance of trends in annual percentage change [9].

The APC model provides adjusted longitudinal age-specific rates for the referent period, reflecting period effects, along with age-specific relative risks of DALYs for each age group to capture age effects. Relative risk is determined by comparing age-specific rates of each group to those of the reference group. Both the age and period rate ratio curves incorporate the entire value of the net drift. Selecting a specific age group or time period as a reference is arbitrary and does not influence the results' interpretation [9].

**S3. Decomposition analysis**

We conducted a decomposition analysis across different SDI provinces to assess the particular effects of socioeconomic and epidemiological changes on the rise in HB-LC stress between 1990 and 2021. This analysis examined the relative impacts of population aging, growth, and epidemiological shifts on age-specific rates. In addition to providing insight into the driving forces behind HB-LC trends in regions with varying economic growth, the breakdown technique enables us to remove each factor's effect while keeping others regular.

**S4. Nordpred analysis for future projections**

We used the Nordpred model to estimate global problem of HB-LC for people over 70 until 2044. This type uses an age-period-cohort framework with an energy link function to make long-term cancer predictions [10]. Projections were stratified by 5-year age cohorts (70-74, 75-79, 80-84, 85-89, 90-94, and 95+) and gender (male and female). The analysis focused on two key indicators: the percentage of HB-LC cases and the DALY charges per 100, 000 people. International projections for 2021 to 2044 were based on empirical data from 1990 to 2020 and were fitted into the design. To provide more conservative long-term projections, the default characteristics of the Nordpred model were a power of 5 for the link work and a gradual decline of the slide part by 25 % in the second and 50 % in the third five-year time.

**References**

1. C. Maher, G. Ferreira, Time to reconsider what Global Burden of Disease studies really tell us about low back pain, Ann Rheum Dis, 81 (2022) 306-308.
2. F. Cao, Y.C. Liu, Q.Y. Ni, Y. Chen, C.H. Wan, S.Y. Liu, L.M. Tao, Z.X. Jiang, J. Ni, H.F. Pan, Temporal trends in the prevalence of autoimmune diseases from 1990 to 2019, Autoimmun Rev, 22 (2023) 103359.
3. X. Qu, M. Liu, C. Ke, J. Liang, Y. Du, L. Yao, J. Li, G. Mu, S. Liu, C. Wang, Burden of alcohol use disorders in China and the regions with different income levels over the world, J Glob Health, 11 (2021) 08011.
4. H.J. Kim, M.P. Fay, E.J. Feuer, D.N. Midthune, Permutation tests for joinpoint regression with applications to cancer rates, Stat Med, 19 (2000) 335-351.
5. S. Kim, S. Lee, J.I. Choi, H. Cho, Binary genetic algorithm for optimal joinpoint detection: Application to cancer trend analysis, Stat Med, 40 (2021) 799-822.
6. P.S. Rosenberg, W.F. Anderson, Age-period-cohort models in cancer surveillance research: ready for prime time?, Cancer Epidemiol Biomarkers Prev, 20 (2011) 1263-1268.
7. A. Bell, Age period cohort analysis: a review of what we should and shouldn't do, Ann Hum Biol, 47 (2020) 208-217.
8. P.S. Rosenberg, D.P. Check, W.F. Anderson, A web tool for age-period-cohort analysis of cancer incidence and mortality rates, Cancer Epidemiol Biomarkers Prev, 23 (2014) 2296-2302.
9. J.J. Yang, E.M. Trucco, A. Buu, A hybrid method of the sequential Monte Carlo and the Edgeworth expansion for computation of very small p-values in permutation tests, Stat Methods Med Res, 28 (2019) 2937-2951.
10. B. Moller, H. Fekjaer, T. Hakulinen, H. Sigvaldason, H.H. Storm, M. Talback, T. Haldorsen, Prediction of cancer incidence in the Nordic countries: empirical comparison of different approaches, Stat Med, 22 (2003) 2751-2766.
